# An analysis of how health systems integrated priority-setting in the pandemic planning in a sample of Latin America and the Caribbean countries

**DOI:** 10.1186/s12961-022-00861-y

**Published:** 2022-05-31

**Authors:** Claudia-Marcela Vélez, Bernardo Aguilera, Lydia Kapiriri, Beverley M. Essue, Elysee Nouvet, Lars Sandman, Iestyn Williams

**Affiliations:** 1 Department of Health, Aging & Society, McMaster University, 1280 Main Street West, Kenneth Taylor Hall Room 226, Hamilton, ON L8S 4M4 Canada; 2 Faculty of Medicine, University of Antioquia, Cra 51d #62-29, Medellín, Antioquia Colombia; 3 Facultad de Medicina y Ciencia, Universidad San Sebastian, Providencia, Santiago, Región Metropolitana Chile; 4 Centre for Global Health Research, St. Michael’s Hospital, 30 Bond St, Toronto, ON M5B 1W8 Canada; 5 School of Health Studies, Western University, 1151 Richmond Street, London, ON N6A 3K7 Canada; 6 National Centre for Priorities in Health, Department of Health, Medicine and Caring Sciences, Linköping University, 581 83 Linköping, Sweden; 7 Health Services Management Centre, University of Birmingham, 40 Edgbaston Park Rd, Birmingham, B15 2RT United Kingdom

**Keywords:** Priority-setting, COVID-19, Pandemic plans, Evaluation, Latin America and Caribbean countries

## Abstract

**Background:**

Latin America and the Caribbean (LAC) are among those regions most affected by the COVID-19 pandemic worldwide. The COVID-19 pandemic has strained health systems in the region. In this context of severe healthcare resource constraints, there is a need for systematic priority-setting to support decision-making which ensures the best use of resources while considering the needs of the most vulnerable groups. The aim of this paper was to provide a critical description and analysis of how health systems considered priority-setting in the COVID-19 response and preparedness plans of a sample of 14 LAC countries; and to identify the associated research gaps.

**Methods:**

A documentary analysis of COVID-19 preparedness and response plans was performed in a sample of 14 countries in the LAC region. We assessed the degree to which the documented priority-setting processes adhered to established quality indicators of effective priority-setting included in the Kapiriri and Martin framework. We conducted a descriptive analysis of the degree to which the reports addressed the quality parameters for each individual country, as well as a cross-country comparison to explore whether parameters varied according to independent variables.

**Results:**

While all plans were led and supported by the national governments, most included only a limited number of quality indicators for effective priority-setting. There was no systematic pattern between the number of quality indicators and the country’s health system and political contexts; however, the countries that had the least number of quality indicators tended to be economically disadvantaged.

**Conclusion:**

This study adds to the literature by providing the first descriptive analysis of the inclusion of priority-setting during a pandemic, using the case of COVID-19 response and preparedness plans in the LAC region. The analysis found that despite the strong evidence of political will and stakeholder participation, none of the plans presented a clear priority-setting process, or used a formal priority-setting framework, to define interventions, populations, geographical regions, healthcare setting or resources prioritized. There is need for case studies that analyse how priority-setting actually occurred during the COVID-19 pandemic and the degree to which the implementation reflected the plans and the parameters of effective priority-setting, as well as the impact of the prioritization processes on population health, with a focus on the most vulnerable groups.

**Supplementary Information:**

The online version contains supplementary material available at 10.1186/s12961-022-00861-y.

## Background

Latin America and the Caribbean (LAC) stand among those regions most affected by the COVID-19 pandemic, exceeding the average number of reported cases and deaths globally and in other regions, including Europe and North America [1–3]. As of 6 October 2021, five of the world’s 20 countries with the highest reported COVID-19 cases and deaths throughout the pandemic were LAC countries (Brazil, Colombia, Argentina, Mexico and Peru). The region faces clear challenges in its ongoing response and continued recovery efforts. These include high levels of inequalities, pre-existing social discontent, low trust between government and the public, high rates of informal employment, weak social protection, fragmented health systems, low economic growth, and inconsistent and changing plans on how to limit community transmission [4]. The current situation has been described as syndemic, whereby previously marginalized and vulnerable communities, social groups and people are at greater risk of infection, complications or death from COVID-19 [5–7]. Furthermore, the COVID-19 pandemic has imposed a double burden on the health systems of some LAC countries, where other tropical diseases such as malaria and dengue coexist, and require public health interventions and epidemiological surveillance alongside COVID-19 [8].

The pandemic has had very high social and economic costs for the region by increasing population poverty and socioeconomic inequalities. It has also exposed the weaknesses of the health and social protection systems, which have mainly impacted the most vulnerable populations. The preparedness of countries in LAC has also been undermined by political factors. For example, some political leaders have repeatedly minimized the threat of COVID-19 by not transparently communicating accurate data regarding the number of deaths and infections within their countries [9], while others have fostered a (false) dilemma between saving the economy or lives, motivated by a “political and economic agenda that disregards the effects of the pandemic in humanitarian terms” [10].

The COVID-19 emergency has strained health systems and shifted critical health resources from routine programmes towards containing the spread of the pandemic and treating those that fall seriously ill [11, 12]. In this difficult scenario, health policy-makers from LAC must determine how to allocate the meagre resources among competing interventions, populations, healthcare settings, and geographical regions [13, 14]. In addition, local authorities have to make decisions as to which groups to prioritize for care, including prioritizing groups for ventilators, personal protective equipment (PPE), therapeutics and vaccines [15]. In these contexts of severe resource constraint, there is a need for systematic priority-setting so that urgent decisions and actions are taken in ways that make the best use of resources, address the primary areas of concern, and avoid harming the most vulnerable groups [10, 16].

The COVID-19 context provides a unique scenario to study and understand whether and how priority-setting was undertaken in a sample of LAC during pandemic times. As part of a global study,^1^ the present paper offers a synthesis of how priority-setting concepts were incorporated into COVID-19 preparedness plans in a variety of LAC countries. Focusing on one region permitted some assumptions about shared characteristics and challenges faced by countries in the region. The aim of this paper is to provide a critical description and analysis of the priority-setting considered in the response and COVID-19 preparedness plans of a sample of LAC. A secondary objective is to explore the degree to which the documented priority-setting processes adhere to established quality indicators of effective priority-setting [17].

## Methods

Analysis for this paper is based on a review of COVID-19 response and preparedness planning documents [18]. Kapiriri and Martin’s framework guided the study through the assessment of the quality parameters of healthcare priority-setting [17] (see Table 1). Specifically, we present the analysis of response and preparedness planning documents from the LAC countries.

**Table 1:** Kapiriri and Martin’s framework for assessing the quality of priority-setting

Domain	Parameter	Short definition
Contextual factors	Conducive political, economic, social and cultural context	^1^Relevant contextual factors that may impact priority-setting
Prerequisites	Political will	Degree to which the politicians manifested the support to tackle the pandemic
	Resources	Availability of a budget in the COVID-19 plan, and clear description of resources available or required (including human resources, ICU beds and equipment, PPE and other resources)
	Legitimate and credible institutions	Degree to which the priority-setting institutions can set priorities, public confidence in the institution
	Incentives for compliance	Explicit description of material and financial incentives to comply with the pandemic plan
The priority-setting process	Planning for continuity of care across the health systems	^2^Explicit mentions of the continuity of healthcare services during the pandemic
	Stakeholder participation	Description of stakeholders participating in the development and implementation of the COVID-19 plan
	Use of clear priority-setting processes/tools/methods	Documented priority-setting process and/or use of priority-setting framework
	Use of explicit relevant priority-setting criteria	Documented/articulated criteria for priority-setting in the COVID-19 plan
	Use of evidence	Explicit mention of the use of evidence to understand the context, the epidemiological situation, or to identify and assess possible interventions to be implemented
	Reflection of public values	Explicit mention that the public is represented or that public values have been considered for the development or implementation of the plan
	Publicity of priorities and criteria	Evidence that the plan and criteria for priority-setting have been publicized and documents are openly accessible
	Functional mechanisms for appealing the decision	Description of mechanisms for appealing decisions related to the COVID-19 plan, or evidence that the plan has been revised
	Functional mechanisms for enforcement the decision	Description of mechanisms for enforcing decisions related to the COVID-19 plan
	Efficiency of the priority-setting process	^3^Proportion of meeting time spent on priority-setting; number of decisions made on time
	Decreased dissensions	^3^Number of complaints from stakeholders
Implementation	Allocation of resources according to priorities	Degree of alignment of resource allocation and agreed upon priorities
	Decreased resource wastage/misallocation	^3^Proportion of budget unused, drug stock-outs
	Improved internal accountability/reduced corruption	Description of mechanisms for improving the internal accountability or reduce corruption
	Increased stakeholder understanding, satisfaction and compliance with the priority-setting process	^3^Number of stakeholders attending meetings, number of complaints from stakeholders, % stakeholders that can articulate the concepts used in priority-setting and appreciate the need for priority-setting
	Strengthening of the priority-setting institution	^3^Indicators relating to increased efficiency, use of data, quality of decisions, and appropriate resource allocation, % stakeholders with the capacity to set priorities
	Impact on institutional goals and objectives	^3^% of institutional objectives met that are attributed to the priority-setting process
Outcome/impact	Impact on health policy and practice	Changes in health policy to reflect identified priorities and swiftness of the pandemic response
	Impact on population health	Description of the expected impact of the COVID-19 plan on the population health
	Impact on reducing inequalities	Description of the expected impact of the COVID-19 plan on reducing inequalities
	Fair financial contribution	Description of the expected impact of the COVID-19 plan on fair financial contributions
	Increased public confidence in the health sector	Description of the expected impact of the COVID-19 plan for increasing public confidence in the response to the COVID-19 pandemic
	-Responsive healthcare system	^3^% reduction in DALYs, % reduction of the gap between the lower and upper quintiles, % of poor populations spending more than 50% of their income on healthcare, % users who report satisfaction with the healthcare system
	Improved financial and political accountability	^3^Number of publicized financial resource allocation decisions, number of corruption instances reported, % of the public reporting satisfaction with the process
	Increased investment in the health sector and strengthening of the healthcare system	^3^Proportion increase in the health budget, proportion increase in the retention of health workers, % of the public reporting satisfaction with the healthcare system

*DALYs* disability-adjusted life-years, *ICU* intensive care unit

^1^This parameter was not assessed in the national COVID-19 plans, but the information about the political, economic, social and cultural context was obtained from different sources and provided in this study to identify similarities and differences among countries in the same region

^2^This parameter was added to the framework for the specific context of the COVID-19 pandemic

^3^This parameter was not possible to be assessed in the national COVID-19 plans

### Countries sampled

Fourteen of the 33 LAC countries were sampled for maximum variation with respect to the following: regional representation (north, central, south and the Caribbean); economic status (World Bank current 2020 fiscal year criteria^2^); political system (presidential republic, federal presidential, or parliamentary republic); health system (public/private, universal/blended); and experiences with prior disease outbreaks. All the sampled countries had prior experience with healthcare priority-setting, whereby they employed various priority-setting approaches to develop their health benefit packages (Table 2).^3^^,^^4^

**Table 2:** Priority-setting context by country

Economic status	Country	Geographical region	Political system	Health system financing (public, private, mixed)	Type of health system (UHC or not)	UHC service coverage index	Total health expenditure per capita in PPP-2018^a^ (USD)	Gini index	Experience with outbreaks
High	Bahamas	Caribbean	Parliamentary democracy under a constitutional monarchy	Mixed public–private (private insurance)	UHC	75	2.005		Influenza
	Chile	South America	Presidential republic	Mixed public–private (public insurance and private insurance)	UHC	70	2.305	46.6	Influenza
	Panama	Central America	Presidential republic	Mixed public–private (private insurance)	UHC	79	1.856	49.9	Influenza
Upper middle	Argentina	South America	Presidential republic	Social security mixed with private (out-of-pocket and private health insurance)	UHC	76	1.989	40.6	Influenza
	Brazil	South America	Federal presidential republic	Social security mixed with private (out-of-pocket and private health insurance)	UHC	79	1.530	50.3	Influenza Zika Chikungunya Dengue Yellow fever
	Colombia	South America	Presidential republic	Social security mixed with private (out-of-pocket and private health insurance)	UHC	76	1.155	49.7	Influenza Zika Chikungunya Dengue Yellow fever
	Dominican Republic	Caribbean	Presidential republic	Social security mixed with private (out-of-pocket and private health insurance)	UHC	74	1.017	45.7	Influenza Zika Chikungunya Dengue
	Mexico	North America	Federal presidential republic	Social security mixed with private (out-of-pocket and private health insurance)	UHC	76	1.154	43.4	Influenza
	Paraguay	South America	Presidential republic	Mixed public–private (out-of-pocket, private insurance)	No UHC	69	935	48.8	Influenza Zika Chikungunya Dengue
	Peru	South America	Presidential republic	Mixed public–private (private insurance)	UHC	77	766	43.3	Influenza Zika Chikungunya Dengue Yellow fever
Lower middle	Bolivia	South America	Presidential republic	Mixed public–private (out-of-pocket, private insurance, donors)	UHC	68	496	44	Influenza Chikungunya
	El Salvador	Central America	Presidential republic	Mixed public–private (out-of-pocket, private insurance, donors)	UHC	76	592	38	Influenza
	Haiti	Caribbean	Semi-presidential republic	Mixed public–private (out-of-pocket, private insurance, donors)	No UHC	49	143	41.1	Influenza Zika Chikungunya Dengue
	Honduras	Central America	Presidential republic	Mixed public–private (out-of-pocket, private insurance, donors)	No UHC	65	362	50.3	Influenza

*PPP* purchasing power parity, *UHC* universal health coverage

^a^International US dollars. Not inflation-adjusted

### Document retrieval and review

#### Search strategy

Two trained members of the research team conducted the document search between August and December 2020. They initially accessed the webpages of the ministries of health and official government websites for the selected countries. The full list of websites consulted is available in Additional file 1. As some preparedness plans were unavailable on government websites, we conducted additional searches in Google and Google Scholar to identify documents. In cases where we were unable to locate a single COVID-19 response and preparedness planning document, we systematically searched for relevant documents (e.g. searching the country’s COVID-19 response website), and emailed contacts of the research team within the country or region for guidance in the process of identification and retrieval of pandemic plans.

#### Document selection

We included all documents that contained COVID-19 response and preparedness plans. In most cases, this was a single, general national COVID-19 response and preparedness plan; in other instances, details of the government response plan were dispersed over multiple documents (see Additional file 1). Two researchers conducted an initial scan of the documents to ascertain their relevance. Documents that covered information on the mobilization and allocation of resources for health services were included. Documents that focused on general government response (e.g. sustaining the economy) or other specific services (e.g. school closures) were excluded. We used native language speakers (Spanish, Portuguese and French) to screen and review any documents that were not written in the English language.

### Data extraction

Data extraction was guided by Kapiriri and Martin’s framework for assessing the quality of healthcare priority-setting in low-income countries [17]. The quality parameters within the framework developed by Kapiriri and Martin were identified through a review of the literature on best practices in priority-setting and interviews with priority-setting experts. The Kapiriri and Martin framework was validated at the global level and has been used to evaluate priority-setting in different health programmes, including disease outbreaks [19]. The framework is comprised of five domains with 26 quality parameters, made up of the priority-setting context (six parameters), prerequisites (four parameters), the priority-setting process (eight parameters), implementation (two parameters) and impact (six parameters) (see Table 1 for a description the parameters). Given how the COVID-19 pandemic has highlighted existing health inequities, we also looked at equity considerations. Assuming equitable priority-setting should take into account the needs of the most vulnerable people, we specifically looked at whether and how vulnerable populations are identified and/or prioritized in the plans [20]. This overall framework provided a consistent standard against which the plans were assessed.

In a prior project, a data extraction tool based on the 20 quality indicators of effective priority-setting was developed and used to evaluate priority-setting during disease outbreaks in Uganda [16]. This formed the basis for developing the data extraction tool. Given the particular impact of the COVID-19 pandemic on health resources at all levels, the research team deemed it important to add specific parameters on level of resource scarcity, the resources identified, priority-setting for health research, and plan for continuity of care across the health system. The revised tool was pilot tested by two research team members who met to compare their outputs following review of the same two preparedness plans and to ensure consistency in their interpretation and application of the revised tool. Only following this pilot testing for consistency and completeness was the tool deemed final and used for data extraction from all preparedness plans. Another team member who was not involved in the initial extraction independently reviewed and validated the extracted information against the original documents to ensure further consistency.

### Data analysis

The initial analysis was descriptive, involving assessment of the degree to which the reports addressed the quality parameters for each individual country. This provided an understanding of the aspects of priority-setting considered within the different settings. The second level of analysis involved a more detailed assessment of the content of each of the parameters, based on the available information; for example, for stakeholder involvement we described the stakeholders identified. The last level of analysis involved a cross-country comparison. The purpose was to assess which countries had the greatest number of parameters and whether this varied according to the dependent variables.

A principal component analysis of Rasch residuals (PCAR), a technique which uses the dependencies between the variables to represent it in a more tractable, lower-dimensional form, without losing too much information, was performed [21]. PCAR is one of the simplest and most robust ways of doing a dimensionality reduction for identifying the common factor that explains similarities and differences among the countries [21–24]. A Wright map was used to present, on the same logit scale, how likely (or how less likely) it was for the different parameters to be identified in the reviewed pandemic plans [24, 25]. The statistical analysis was performed with Winsteps 3.65 [26].

## Results

A total of 14 national COVID-19 preparedness and response plans were included in this study (about 40% of all LAC countries). Of the 14, 12 plans were in the form of a single national COVID-19 pandemic, while for two countries (Chile and Mexico), several documents collectively comprised the countries’ COVID-19 pandemic plan. All documents were published between February 2020 and July 2020.

The study sample included four low- to middle-income countries (Bolivia, El Salvador, Haiti, Honduras), seven upper- to middle-income countries (Argentina, Brazil, Colombia, Dominican Republic, Mexico, Paraguay and Peru) and three high-income countries (Bahamas, Chile, and Panama). The countries were at different stages of the COVID-19 pandemic at the time of retrieval.

**Fig. 1:**
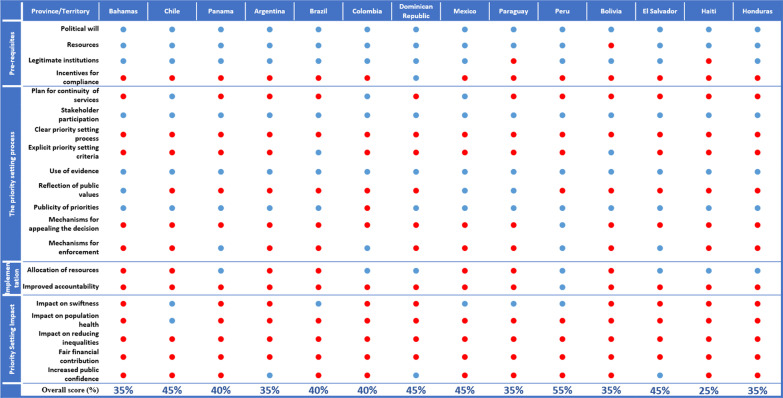
Country performance on priority-setting parameters according to the plans accessed

In what follows, we describe results organized according to the five domains of priority-setting, as identified in Kapiriri and Martin’s framework, including priority-setting context, prerequisites, priority-setting process, implementation and outcomes/impact. For each domain, and related parameters, we describe the number of countries that included the parameter in their reports, provide a snapshot of their content, and present a cross-country comparison (Fig. 1).

### Priority-setting contexts

The framework identifies five relevant contextual factors: the social, economic, cultural, political and health system contexts. While the reviewed COVID-19 plans did not describe the contexts in detail, the government webpages and the literature were used to obtain the additional information on the priority-setting context. We discuss some of the relevant contextual factors below.

High inequality rates characterize the sociopolitical context of the region. In the last 6 years, many countries have suffered from political and social instability, in particular Ecuador, Peru, Chile, Bolivia, Colombia and Brazil [27, 28]. These countries have faced waves of social protests that have highlighted long-standing problems in the region: corruption, weak institutions, the rejection of traditional political parties, poverty, insecurity and inequality [27, 28]. Furthermore, all of the vulnerable groups identified in a recent United Nations report on human rights during the COVID-19 pandemic are present in the sampled countries [29]. For example, Bolivia, Guatemala and Mexico are among the 15 countries with the highest proportion of indigenous peoples in their total population [30]. In recent years, the region has also been struggling with mass migration, mainly from Venezuela, whose political and economic instability has caused one of the greatest displacement crises in the world. Millions of Venezuelan migrants and refugees have moved through the region, mostly to Colombia, Peru, Chile and Ecuador [31]. Furthermore, most countries in the LAC region have a large number of people living with HIV, Brazil being the country with the highest number (920 000 persons in 2019) [32].

All the health systems of the sampled countries have a mix of public and private financing, and 13 are considered as having universal health coverage (UHC).

### Prerequisites

The framework identifies four prerequisites for effective priority-setting: political will, availability of a legitimate priority-setting institution, incentives for compliance, and human and financial resources. While the plans discussed political will and resources, and identified the committees that developed the plans, there was neither explicit discussion of the legitimacy and capacity of the committees or priority-setting institutions, nor identification of (dis)incentives for compliance. We discuss the parameters that were presented below.

In relation to *political will*, all the countries provided a response and preparedness plan led by their governments, often with participation of different governmental and social institutions (e.g. medical societies). Ten out of 14 countries (Bahamas, Brazil, Chile, Colombia, the Dominican Republic, El Salvador, Honduras, Panama, Paraguay and Peru) created COVID-19 task forces, inter-ministerial committees or governmental commissions responsible for advising the government and designing the implementation of different interventions.

Although all the countries we sampled had national pandemic influenza plans, only Panama and the Bahamas made an explicit reference that the COVID-19 plan incorporated some of the institutional legacies of previous coordination structures established to respond to the influenza pandemic.

#### Resources

**Fig. 2:**
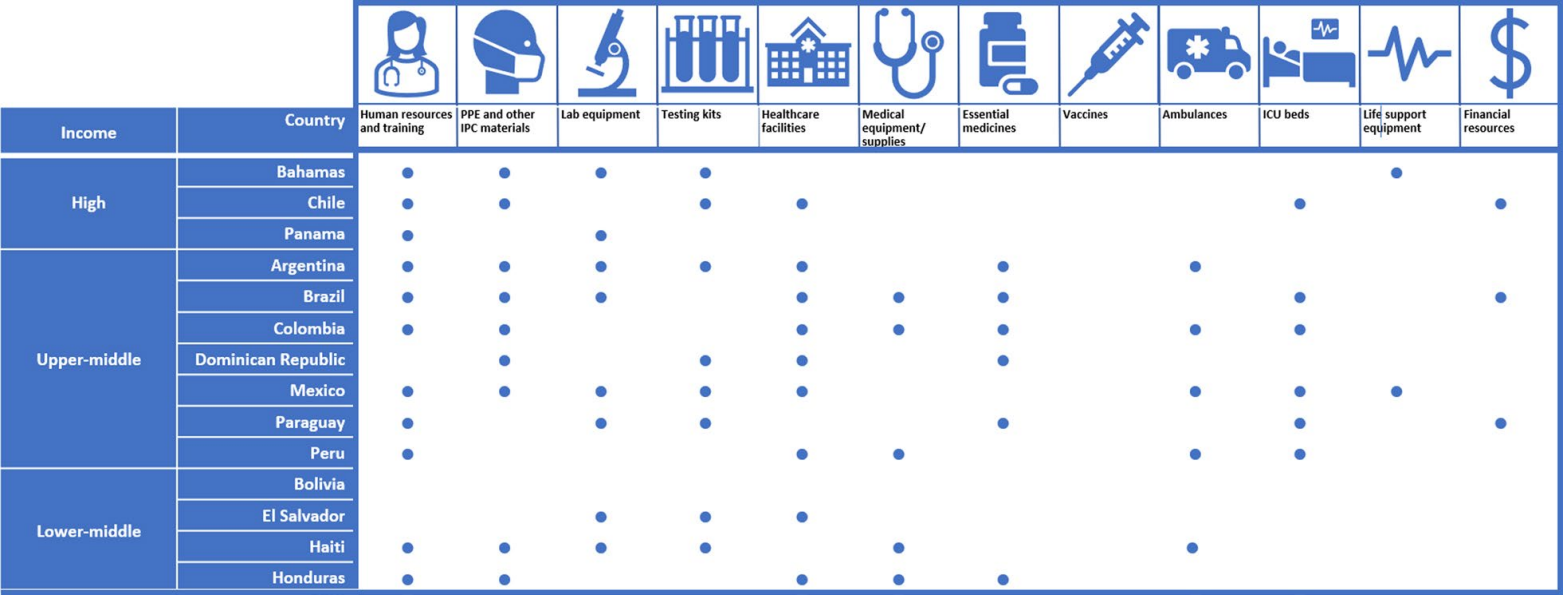
Resource gaps identified

This parameter assesses the availability (or lack) of financial, material and human resources to implement the identified priorities. Apart from Bolivia, all countries’ plans discussed resources relevant to managing COVID-19, identifying PPE and other infection prevention and control (IPC) materials as well as the need for laboratory equipment and specimen transportation (Fig. 2). However, several plans discussed insufficient human resources (11 countries), lack of healthcare facilities (nine countries), insufficient intensive care unit (ICU) beds (six countries), lack of PPE and other IPC materials (nine countries), low testing capacity (eight countries) and insufficient medicines/supplies (seven countries). Notably, while several of the sampled national plans identified insufficient ICU beds, only the Mexico plan mentioned the scarcity of life support equipment.

Since most of the committees that were involved in developing the plans were appointed by the country’s governments, their legitimacy could be inferred by virtue of their appointment. However, it was not possible to assess the committees’ capacity to set priorities and whether there was an increase in the public’s confidence in the institution.

### The priority-setting process

The priority-setting process domain assesses whether the stipulated prioritization process was based on an explicit guiding tool/method/framework, was evidence-based and was fair. Fairness was assessed based on whether the plan articulated explicit priority-setting criteria (including equity considerations), stakeholder involvement, a publicity strategy, and mechanisms for appeals and revisions. Although none of the plans presented clear priority-setting process/tool/methods, several explicitly mentioned the incorporation of the WHO recommendations. For example, the Dominican Republic, Honduras and Panama used the WHO strategy of planning according to three pandemic phases and four scenarios—which do not incorporate priority-setting.

In relation to *prioritizing the sustenance of routine essential services*, Mexico’s plan explicitly mentioned the necessity and importance of the continuity of maternal and child healthcare essential services, providing a plan to ensure it. In Chile, a planning document established that from the 85 diseases already prioritized through a national health benefits package called Universal Access with Explicit Guarantees (AUGE by the acronym in Spanish), only a subset of those diseases (especially neoplasms) would keep their priority status during the pandemic.

In relation to *stakeholder participation*, all plans were led by the ministries of health (or equivalent), and many plans identified intersectoral committees. Commonly identified sectors included agriculture, transportation, education, tourism and finance. Additional stakeholders included medical associations and colleges, representatives of the public and private healthcare institutions, insurance companies, and journalists and media groups. Three plans (Bahamas, Mexico and Paraguay) explicitly indicated that the public was represented or that public values had been considered for the development or implementation of the plan.

Some countries included representatives of WHO in the task forces/commissions/committees coordinating the COVID-19 response plan.

All the country plans were in some way (and to a varied extent) “evidence-informed”. Countries used various forms of evidence, for example, epidemiological data, lessons learnt from previous outbreaks or evidence on effective interventions. Many countries made explicit mention of using WHO pandemic planning guidelines (Bolivia, Brazil, Colombia, Dominican Republic, El Salvador, Haiti, Honduras, Panama and Paraguay), and three countries specifically adopted the WHO planning strategy according to three pandemic phases and four scenarios (Dominican Republic, El Salvador and Honduras).^5^ The plans of the Bahamas and Brazil explicitly mentioned the use of past pandemic plans. Brazil referred to the Middle East respiratory syndrome coronavirus (MERS-CoV) response plan, and Panama and the Bahamas made explicit mention of previous pandemic influenza plans. The Bahamas used the “Influenza and other Vaccine-Preventable Diseases National Emergency Management Agency Plan” and several previous emergency and infectious disease contingency plans.

Only the COVID-19 response and preparedness plan of Brazil articulated *explicit priority-setting principles and/or criteria*. In this plan, the identified criteria included disease transmissibility, geographical spread, clinical severity of the disease, population vulnerability, and availability of preventive measures such as vaccines and possible treatments.

In addition to the general criteria, we assessed whether the plans explicitly identified *equity* as a priority-setting criterion, including whether equity was considered when identifying programmes that should be sustained during the pandemic, and noting strategies proposed to provide healthcare services to vulnerable populations (Table 3).

**Table 3:** Summary of prioritized population groups within the country plans

Criteria for prioritization	Population prioritized	Bahamas	Chile	Panama	Argentina	Brazil	Colombia	Dominican Republic	Mexico	Paraguay	Peru	Bolivia	El Salvador	Haiti	Honduras
Prioritized given the greatest risk of becoming infected and seriously ill	Elderly		**●**				**●**					**●**			
	People immune-compromised											**●**			
	People with comorbidities or predisposing conditions											**●**			
Prioritized given their risk of transmission	Healthcare workers				**●**										
	Travellers		**●**												
	People living in institutions								**●**						
Prioritized given their vulnerability	Racial and ethnic minorities														
	Religious minorities														
	Migrants								**●**						
	Refugees/internally displaced persons (IDPs)														
	Indigenous peoples	NA												NA	
	Persons with disabilities	●							**●**		**●**				
	Prisoners, detainees, and those deprived of their liberty														
	LGBTI people								**●**						
	People living with HIV														
	People who use drugs														
	People with lower socioeconomic status														
	Population in rural areas														
	Homeless population														
Prioritized for continuity of services	Pregnant women								**●**						
	Young infants								**●**						
	People in need of sexual and reproductive services														
	People with pre-existing illnesses														
	People living with HIV														

*LGBTI* lesbian, gay, bisexual, transgender, and intersex

Only three plans (Bahamas, Mexico and Peru) identified vulnerable populations and explicitly prioritized them in the COVID-19 planning. The vulnerable populations included people with disabilities (all three countries), immigrants, and sexual and gender minorities (Mexico). Only the Mexico and Chile plans considered the continuity of services for pregnant women and young infants, or people with particular diseases, respectively.

In addition to the priority-setting criteria and equity considerations, the Kapiriri and Martin framework requires that priority-setting decisions and their rationales be made *publicly available*, and recommends the presence of mechanisms for *publicizing, appealing and revising* the decisions as well as *mechanisms for enforcing* the three prior conditions. While nine plans included a section on communication, this was mainly risk communication, oriented towards health education and not information about priority-setting decisions. However, all the countries developed webpages to communicate decisions and to provide citizens information about the unfolding pandemic. While there were no clear appeals or revisions mechanisms, five plans (Peru, Mexico, Brazil, Chile and Paraguay) mentioned that the established commissions/task forces would periodically review additional emerging national and/or international evidence and use it to revise their plans and activities. Some countries tasked certain stakeholders (ministries of health or regional health authorities) with the role of evaluating or supervising compliance with the plan (Argentina, Colombia, El Salvador and Panama).

*Mechanisms for appealing the decisions* were only considered in the preparedness and response plan for Peru, which highlighted that laws could be revised and decisions might change according to the dynamic epidemiological conditions. Enforcement mechanisms to ensure that the process adhered to the above conditions were not articulated. However, some plans (Panama, Colombia, El Salvador and Peru) articulated mechanisms for ensuring that the decisions were implemented. These mostly referred to the proclamation of laws that enforced the compliance with lockdowns, social distancing and restrictions on gatherings and public transportation.

There were no discussions about the efficiency of the prioritization process or the reduction in public dissensions.

### Implementation of the set priorities

This domain assesses the *degree to which resources were allocated according to the priorities*, *reduction in resource wastage*, *improved accountability and reducing corruption*, *increased stakeholder understanding of priority-setting and strengthening of the priority-setting institutes*.

Some countries specified the need for reallocation of resources (monetary and others) from less prioritized areas to high-priority areas (Colombia, Dominican Rep, Haiti, Honduras, Panama and Peru).

While several plans included budget estimates for the pandemic priorities, only Peru’s plan talked about aiming to improve internal accountability/reduce corruption by supporting transparency in the management of resources and accountability.

The other parameters were not discussed in the reviewed documents.

### Impact and outcome

This domain has the following parameters: *impact on institutional goals and objectives, impact on health policy and practice, impact on population health, impact on reducing inequalities, fair financial contribution, increased public confidence in the health sector, responsive healthcare system, improved financial and political accountability, increased investment in the health sector and strengthening of the healthcare system.*

All these parameters could not effectively be assessed from the information in the planning documents. However, some of the plans indicated that they would mobilize additional resources for financing the plan, and the need to strengthen the healthcare system to respond to the pandemic. Since these parameters can only be effectively assessed after the plans are implemented, interviews with decision makers and implementers to evaluate the degree to which these parameters were actually achieved.

### Cross-country comparison

The PCAR enabled us to identify patterns between countries in relation to the number of parameters contained within their plans. According to the Wright mapping, all the sampled country plans included only a few parameters (the red box). When assessing which country plans identified the various parameters (side A of the figure), the countries at the top of the figure are those whose plans identified the greatest number of parameters (including those that were least likely to be found in the other plans). When assessing the likelihood that the parameters were found in the plans (side B of the figure), parameters above zero (e.g. incentives for compliance and mechanisms for appealing the decisions) were the least likely to be found in the reviewed plans, while those below zero (e.g. stakeholder engagement) were more likely to be found in the reviewed plans.

**Fig. 3:**
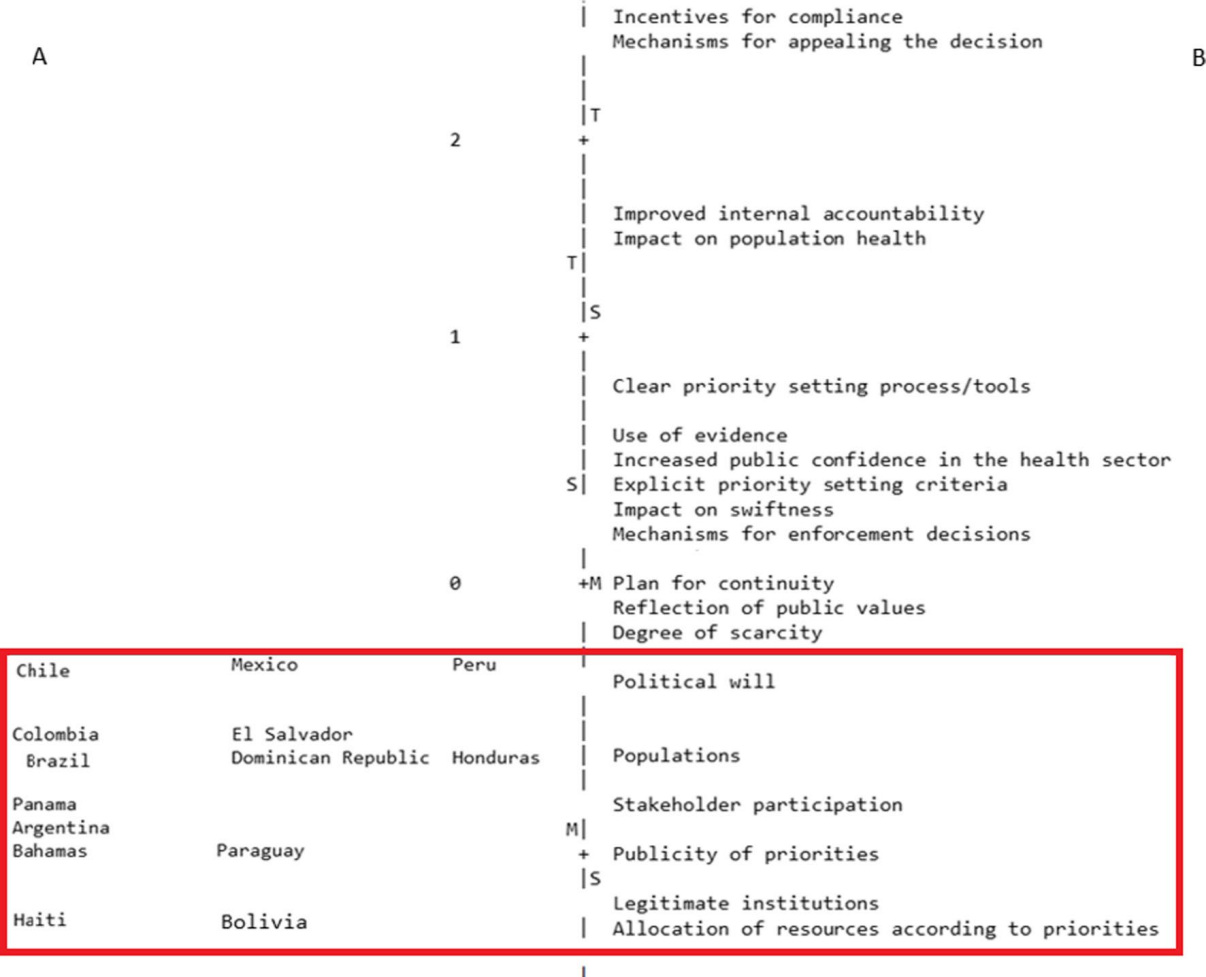
Wright map. The figure has two sides separated by a punctuated line. Side A has the country plans, and side B the parameters. Parameters over zero indicate they are less likely to be found in the response and preparedness plans; for instance, incentives for compliance and mechanisms for appealing the decisions were the least likely to be found in the reviewed plans. Countries are also ordered by level of trait; the countries on the top of the figure are those whose plans identified the greatest number of parameters, including those that were least likely to be found in the other plans. Overall, the figure shows that all the sampled country plans included only a few parameters (the red box)

Based on this analysis, three groups of countries were identified. The plans from the first group of countries (which included Mexico, Chile, Dominican Republic and El Salvador) contained the highest number (9–11) of parameters. Those from the second group (Argentina, Brazil, Colombia, Honduras, Panama, Paraguay and the Bahamas) contained 7–8 parameters, and the plans from the third and last group (Bolivia and Haiti) had the lowest number (six) of parameters. (Fig. 3). There were no patterns observed between the groupings of the countries according to the PCAR analysis and countries’ experiences with disease epidemics, health system type or political system.

## Discussion

WHO warns that when priority-setting “is not explicitly done, with a transparent discussion on priority-setting criteria and a joint examination of the evidence, then it will be done in an ad hoc, implicit way”.^6^ Similarly, in the context of the current pandemic, the Inter-American Commission on Human Rights (IACHR) has called on countries to develop criteria for priority-setting in a transparent and participatory manner.^7^

Hence, this study provides the first descriptive analysis of whether and how COVID-19 response and preparedness plans in the LAC region included priority-setting. The analysis, based on Kapiriri and Martin’s [17] framework of effective priority-setting, found that while all the reviewed plans included various aspects of priority-setting, none included all the parameters. Many parameters from the priority-setting process domain were not commonly addressed in the plans. We interpreted this finding as a consequence of the lack of incorporation of explicit process and criteria for priority-setting in regular health policy decision-making [33–39], which is supported by many authors, and which might be worsened by the urgency in planning a response to the COVID-19 pandemic [39–42].

Response and preparedness plans in all 14 countries included in this analysis were led by the country’s government. In 10 of these countries, specific COVID-19 task forces, inter-ministerial committees, or governmental commissions were created to coordinate some activities of the plan and/or to advise government. Most plans also acknowledged the participation of civil society actors such as scientific societies and clinical experts.

Despite the strong evidence of political will and stakeholder participation, none of the plans presented a clear priority-setting process, or used a formal priority-setting framework, to define interventions, populations, geographical regions, healthcare setting or resources prioritized. Likewise, most plans did not present explicit guiding criteria or principles for priority-setting. Although the plans of Brazil and Bolivia mentioned some criteria, they included no systematic discussion or application of the criteria in the prioritization process. In this respect, the plans deviate from the WHO and the IACHR recommendations.

All the reviewed plans (except Bolivia) identified availability/scarcity of various resources that were deemed relevant to managing the COVID-19 pandemic. This is consistent with the priority-setting literature, which emphasizes the need for explicit identification of both priorities and resources [20]. The identified resources (human and material healthcare resources, such as healthcare personnel, healthcare facilities, PPE and testing kits) are also consistent with the literature on COVID-19 resource requirements [43]. However, only six national plans identified insufficient ICU beds, and only one mentioned the lack of life support equipment. It is possible that the less attention given to these resources is due to most of the plans having been published early in the pandemic, when the main goal was prevention and the number of cases had not yet resulted in a rapid increase in hospital admissions.

One approach to incorporating equity considerations is by prioritizing vulnerable populations [44]. Our findings that only three countries explicitly included the consideration of various vulnerable populations (as priorities for COVID-19 interventions or continuity of services) could be an indicator of the degree to which these countries accounted for equity in their plans. Although it is possible that after publishing the plans, governments made further adjustments to them, the general lack of explicit prioritization of vulnerable populations is concerning. As Blanchet et al. (2020, p. 2) note, “a decline in supply and demand for non-COVID-19 essential routine health services may exacerbate the general health situation and lead to excess mortality beyond what is directly attributed to the pandemic” [45]. Moreover, the suspension of essential health services is likely to impact the most socially disadvantaged populations first and increase existing inequalities. Unfortunately, this finding is not uncommon. A similar study in the WHO African Region found limited focus on prioritizing the known vulnerable groups in the region [20]; LAC-specific literature also identifies some vulnerable groups relevant to this context [46]. This overall lack of attention to vulnerable populations in particular, and equity considerations more generally, diverges from the guidance of the IACHR, according to which governments should pay particular attention to people in vulnerable situations in their response to the pandemic.^8^

According to the IACHR resolutions on the Pandemic and Human Rights in the Americas of 10 April 2020, and Human Rights of Persons with COVID-19 of 27 July 2020, the rights of certain people or groups, such as indigenous peoples, persons deprived of liberty, people living with HIV/AIDS, and women, among others, may be at special risk during the pandemic. Indeed, some studies in the United States and Latin America have indicated that in vulnerable communities and regions with a higher rate of poverty, unemployment or informal employment, or where ethnic minorities live, there is a higher prevalence of COVID-19 and a higher risk of mortality, regardless of age and the existence of comorbidities [47–51]. This evidence, together with the large presence of vulnerable populations and high levels of socioeconomic inequalities in the LAC region [29–32, 52], indicates that prioritizing vulnerable populations in the COVID-19 plans is not only a humanitarian imperative but also a necessity for reducing the incidence and mortality of the disease.

The purpose of the PCAR cross-country comparison was to assess the degree to which the priority-setting quality parameters in the reviewed plans reflected particular health system organization, pandemic experiences and socioeconomic status. Although the differences between the countries were not substantial, the findings that more lower- and middle-income countries tended to have a smaller number of quality parameters in their plans, and three of them lacked UHC (Haiti, Honduras and Paraguay), highlight a possible link between priority-setting, economic status and the type of health system. While these linkages may not be clear, some of the literature alludes to the weaknesses in priority-setting infrastructure in contexts with weak health infrastructure and limited resources [13].

### Limitations

The study findings should be interpreted with caution for the following reasons: First, the response and preparedness plans reviewed were published at different stages of the global and national COVID-19 pandemic. The comprehensiveness of the plans may have been affected by this temporal difference. Second, for two countries (Chile and Mexico), we included several government documents that formed part of the national response plan, as opposed to a single document. While this approach could have led to favourable comparisons with the countries, the cross-country comparison did not indicate any additional advantage for the two countries. Third, basing the analysis on government documents may have introduced governmental bias. However, governments led the pandemic response efforts in most of the countries.

### Implications for research and health policy

Explicit priority-setting is critical to improving the quality of COVID-19 pandemic preparedness and response. Since most LAC countries lacked explicit reference to the parameters of effective priority-setting, incentivizing the study and use of priority-setting frameworks in the LAC region should be encouraged. Countries in the region are unlikely to deliver equitable healthcare services during disease pandemics, especially for marginalized communities, without explicit priority-setting processes. Those processes must reflect the parameters of effective priority-setting, as discussed in this paper. The Pan American Health Organization and the Center for Global Health Development could support these countries in instituting and strengthening their priority-setting mechanisms.

This study highlights the need for case studies on the formulation, implementation and evaluation of public policies (beyond priority-setting) in the face of health emergencies, based on the experience of the COVID-19 pandemic.

Integrating priority-setting in the pandemic plans is an initial step to implementing systematic priority-setting. However, since the plans may, due to various contextual factors, not be implemented, there is need for cases studies that describe how priority-setting was actually implemented during the COVID-19 pandemic in the LAC and how the implementation compared to what was planned. Such case study will begin to unpack the relevant local and global contextual factors that impact priority-setting, which will contribute to the health policy and priority-setting literature.

## Conclusion

To the best of our knowledge, this is the first paper that systematically analyses the inclusion of priority-setting in COVID-19 pandemic plans in the LAC. Based on the analysis using the parameters for effective priority-setting in Kapiriri and Martin’s [17] framework, all the reviewed plans included various (but not all) aspects of effective priority-setting. The variations were neither aligned with the economic or political systems, nor the type of health system or healthcare financing mechanisms. Overall, however, most of the plans included specific committees that were designated to set priorities, and the participation of civil society actors. However, none of the plans presented a clear priority-setting process or used a formal priority-setting framework, and several lacked explicit guiding criteria or principles for priority-setting.

Since systematic priority-setting is critical to improving the quality of COVID-19 (and future) pandemic preparedness and response, LAC should be supported to better integrate the parameters of effective priority-setting in the pandemic plans and their implementation. The Pan American Health Organization and the Center for Global Health Development could support these countries in instituting and strengthening their priority-setting mechanisms. There should be concurrent efforts to integrate evaluation of actual priority-setting in order to understand the actual impact of the prioritization process on population health, but more specifically on the most vulnerable populations in this region.

## Supplementary Information

**Additional file 1.** Documents reviewed in each country.

## Data Availability

The data associated with this manuscript are included in the submission as an additional file.

## Footnotes

1 The aim of the global study is to evaluate the degree to which national COVID-19 preparedness and response plans incorporated priority-setting concepts. The global study includes a sample of over 60 countries from all six WHO regions. The project analysed and reported findings from the USA and Canada separately due to their unique and highly decentralized health systems.

2 For the current 2021 fiscal year, low-income economies are defined as those with a gross national income (GNI) per capita, calculated using the World Bank Atlas method (US dollars), of $1035 or less in 2019; lower middle-income economies are those with a GNI per capita between $1036 and $4045; upper middle-income economies are those with a GNI per capita between $4046 and $12 535; high-income economies are those with a GNI per capita of $12 536 or more.

3 Inter-American Development Bank Social Protection and Health Division. Giedion U, Bitrán R, Tristao I, eds. Health benefit plans in Latin America: a regional comparison. May, 2014. http://publications.iadb.org/bitstream/handle/11319/6484/Health-Benefit-Plans.pdf?sequence=.

4 Health benefits guarantees in Latin America: equity and quasi-market restructuring at the beginning of the millennium. http://www.cepal.org/publicaciones/xml/1/25601/L717.pdf.

5 The WHO guidelines also highlight the need to use evidence when developing the pandemic plans.

6 http://www.oas.org/es/cidh/decisiones/pdf/Resolucion-1-21-es.pdf.

7 http://www.oas.org/es/cidh/decisiones/pdf/Resolucion-1-21-es.pdf.

8 https://gchumanrights.org/preparedness/article-on/covid-19-health-and-human-rights-the-inter-american-systems-response.html.
