# Correction to: An analysis of how health systems integrated priority-setting in the pandemic planning in a sample of Latin America and the Caribbean countries

**DOI:** 10.1186/s12961-022-00882-7

**Published:** 2022-07-29

**Authors:** Claudia-Marcela Vélez, Bernardo Aguilera, Lydia Kapiriri, Beverley M. Essue, Elysee Nouvet, Lars Sandman, Iestyn Williams

**Affiliations:** 1 Department of Health, Aging & Society, McMaster University, 1280 Main Street West, Kenneth Taylor Hall Room 226, Hamilton, ON L8S 4M4 Canada; 2 Faculty of Medicine, University of Antioquia, Cra 51d #62-29, Medellín, Antioquia, Colombia; 3 Facultad de Medicina y Ciencia, Universidad San Sebastian, Providencia, Santiago, Región Metropolitana Chile; 4 Centre for Global Health Research, St. Michael’s Hospital, 30 Bond St, Toronto, ON M5B 1W8 Canada; 5 School of Health Studies, Western University, 1151 Richmond Street, London, ON N6A 3K7 Canada; 6 National Centre for Priorities in Health, Department of Health, Medicine and Caring Sciences, Linköping University, 581 83 Linköping, Sweden; 7 Health Services Management Centre, University of Birmingham, 40 Edgbaston Park Rd, Birmingham, B15 2RT UK

## Correction to: Health Research Policy and Systems (2022) 20: 58 https://doi.org/10.1186/s12961-022-00861-y

The original publication of this article contained an incorrect affiliation. The incorrect and correct affiliation is shown in this correction article. The original article [1] has been updated.

Incorrect

Faculty of Medicine and Science at the Universidad San Sebastian, Providencia, Santiago de Chile, Región Metropolitana

Correct

Facultad de Medicina y Ciencia, Universidad San Sebastian, Providencia, Santiago, Región Metropolitana, Chile
