# Supplementary material for: An analysis of how health systems integrated priority-setting in the pandemic planning in a sample of Latin America and the Caribbean countries
Source: Health Res Policy Syst. 2022 May 31;20:58. doi: 10.1186/s12961-022-00861-y (PMC9153233; doi:10.1186/s12961-022-00861-y)
Supplement: Supplementary file 1 — Additional file 1. Documents reviewed in each country. [file 12961_2022_861_MOESM1_ESM.docx]

Additional File 1: Documents reviewed in each country

| **Country** | **Document(s) reviewed** |
| --- | --- |
| Bahamas | [Novel Coronavirus (COVID-19). Preparedness and Response Plan- Draft Version 3](https://macdrive.mcmaster.ca/f/0ff2ad43fa224fdea480/) |
| Chile | - [Lineamientos para el Plan de Funcionamiento 2021](https://macdrive.mcmaster.ca/f/6b3b54251f094fb2a96b/) - [Decree Number 42.696](https://macdrive.mcmaster.ca/f/05cc127bb42941fd938e/) - [Protocolo De Coordinación Para Acciones De Vigilancia Epidemiológica Durante La Pandemia Covid-19 En Chile: Estrategia Nacional De Testeo, Trazabilidad Y Aislamiento](https://macdrive.mcmaster.ca/f/ba1f5a9fc8504300b93d/) - [Protocolo de Reprocesamiento de respiradores N95 y FFP2](https://macdrive.mcmaster.ca/f/2719a2e75bab4eac93b5/) - [Sobre algunas medidas para la protección de personal de la salud](https://macdrive.mcmaster.ca/f/baa2a65f588a4c8eaeb3/) - [Decree Number 42.627](https://macdrive.mcmaster.ca/f/05cc127bb42941fd938e/) - [Decree Number 42.621-B](https://macdrive.mcmaster.ca/f/6cc694fc4a154b8cb970/) - [Protocolo De Detección De Viajeros En Pasos Fronterizos Terrestres](https://macdrive.mcmaster.ca/f/03bee41b975c49aebca0/) - [Protocolo De Identificación Y Seguimiento Para Viajeros Que Provienen De Países Con Transmisión Local De COVID-19](https://macdrive.mcmaster.ca/f/74c4f47ee5a44bd49d00/) - [Decree Number 42.591](https://macdrive.mcmaster.ca/f/bb085bbda36e46efbb2e/) |
| Panama | - [Plan de Acción en caso de brote o epidemia COVID-19 en el territorio nacional Panamá](https://macdrive.mcmaster.ca/f/7d574f9811724b8bb723/) - [Plan Operativo Nacional del Sistema de Salud para la Prevención y Control del Nuevo Coronavirus (2019-nCoV)](https://macdrive.mcmaster.ca/f/b3519d11b2274eed8122/) |
| Argentina | - [Plan Operativo de preparación y respuesta al COVID-19 Argentina](https://macdrive.mcmaster.ca/f/15694bd3e16240608ccf/) |
| Brazil | - [Plano de Contingência Nacional para Infecção Humana pelo novo Coronavírus COVID-19](https://macdrive.mcmaster.ca/f/39473fd855164684b87a/) |
| Colombia | - [Plan de acción para la prestación de servicios de salud durante las etapas de contención y mitigación de la pandemia por SARS-Cov-2 (COVID-19)](https://macdrive.mcmaster.ca/f/f71cce9e86364f4d8968/) |
| Dominican Republic | - [Plan de contingencia ante enfermedad por coronavirus (COVID-19)](https://macdrive.mcmaster.ca/f/7a60d5cfb4a042e4952a/) |
| Mexico | - [Plan gradual hacia la nueva normalidad en la Ciudad de México](https://macdrive.mcmaster.ca/f/140dc432c63640869fca/) - [Plan Operativo De Atención A La Población Migrante Ante COVID-19](https://macdrive.mcmaster.ca/f/a0ea8dde5d0948fca15d/) - [Protocolo de actuación para la atención de COVID-19 al interior de Centros Federales de Reinserción Social (CEFERESOS)](https://macdrive.mcmaster.ca/f/e586c0709c674b6f8edd/) - [Recomendación sobre actividad física para personas con discapacidad psicosocial (trastornos de ansiedad) durante la pandemia COVID-19](https://macdrive.mcmaster.ca/f/adecc1e5a74e407a86a6/) - [Recomendaciones para cuidar la salud mental de adultos mayores durante la pandemia COVID-19](https://macdrive.mcmaster.ca/f/90a495c2512541779248/) - [Recomendaciones para la mitigación de COVID-19 en Refugios y Centros de Atención Externa para mujeres que viven violencia extrema, sus hijos e hijas](https://macdrive.mcmaster.ca/f/eabdf88c8c524261b891/) - [Recursos contra la discriminación y la violencia por orientación sexual, identidad o expresión de género en el contexto de la enfermedad COVID-19](https://macdrive.mcmaster.ca/f/7db014f61c8644a190cd/) - [Criterios para las poblaciones en situación de vulnerabilidad que tienen mayor riesgo de desarrollar una complicación o morir por COVID-19 en la reapertura de actividades económicas en los centros de trabajo](https://macdrive.mcmaster.ca/f/0c9b02c374b545a2acd1/) - [Prestación de servicio de traslado aéreo de insumos COVID-19](https://macdrive.mcmaster.ca/f/49d371ae447042618c35/) - [Atención Domiciliaria De Pacientes Sospechosos O Confirmados De Covid 19 Síntomas leves y SIN enfermedades crónicas subyacentes](https://macdrive.mcmaster.ca/f/0c3cb78c26ed46689f05/) - [Convenio marco para la prestación subrogada de servicios médicos y hospitalarios](https://macdrive.mcmaster.ca/f/387e43597dda49ecb303/) - [Recomendaciones para los Centros de Atención Infantil ante la enfermedad COVID-19](https://macdrive.mcmaster.ca/f/1a18e7cf2eeb4c3cb0f5/) - [Limpieza y desinfección de espacios comunitarios durante la pandemia por SARS-CoV-2](file:///C:\Users\clama\Google%20Drive\Prioritty%20setting\Regions\PAHO\Draft\•%09Limpieza%20y%20desinfección%20de%20espacios%20comunitarios%20durante%20la%20pandemia%20por%20SARS-CoV-2) - [Lineamientos para el desarrollo de acciones de búsqueda en campo en el contexto de la pandemia por SARS-CoV-2 (COVID-19) Municipios de la Esperanza](https://macdrive.mcmaster.ca/f/bcdf485b56a7475183cd/) - [Guía Básica para los Sistemas Prehospitalarios para COVID-19](https://macdrive.mcmaster.ca/f/a1a5030e283e44e8a1fa/) - [Guía para la protección de la salud de las personas con discapacidad en el contexto de COVID-19](https://macdrive.mcmaster.ca/f/45e2a4f939ce47888f04/) - [Lineamiento de Reconversión Hospitalaria](https://macdrive.mcmaster.ca/f/d466617036b44295b2b0/) - [Lineamiento para la atención de pacientes por COVID-19](https://macdrive.mcmaster.ca/f/bed31b75beb54cf8b9c4/)[Lineamiento técnico de uso y manejo del equipo de protección personal ante la pandemia por COVID-19](https://macdrive.mcmaster.ca/f/5959ee3567fa4cd6b26d/) - [Lineamiento para la prevención y mitigación de COVID-19 en la atención del embarazo, parto, puerperio, y de la persona recién nacida. Versión 2](https://macdrive.mcmaster.ca/f/d7447197a85045aa9035/) |
| Paraguay | - [Coronavirus (SARS-CoV-2) National Plan of Response to Respiratory viruses 2020 (Coronavirus (SARS-CoV-2) Plan Nacional De Respuesta A Virus Respiratorios 2020)](https://macdrive.mcmaster.ca/f/9aae7445ef0b4d0bb7c0/) |
| Peru | - [Plan Nacional de Reforzamiento de los Servicios de Salud y Contención del COVID 19](https://macdrive.mcmaster.ca/f/d4cac01322b2454486b9/) |
| Bolivia | - [Guía para el Manejo del COVID-19](https://macdrive.mcmaster.ca/f/eab79d3c272c468989ad/) |
| El Salvador | - [Plan Nacional de Preparación y Respuesta al Nuevo Coronavirus (nCov 2019)](https://macdrive.mcmaster.ca/f/2544d2f3ae2b4482b204/) |
| Haiti | - [Plan de préparation et de réponse du MSPP au Coronavirus](https://macdrive.mcmaster.ca/f/727f248a76a24fe2a198/) |
| Honduras | - [Plan para la Contención y Respuesta a Casos de Coronavirus (Covid-19) En Honduras](https://macdrive.mcmaster.ca/f/e78656ef2b6a4d868882/) |
